# Inhibition Underlies the Effect of High Need for Closure on Cultural Closed-Mindedness under Mortality Salience

**DOI:** 10.3389/fpsyg.2016.01583

**Published:** 2016-10-25

**Authors:** Dmitrij Agroskin, Eva Jonas, Johannes Klackl, Mike Prentice

**Affiliations:** Department of Psychology, University of SalzburgSalzburg, Austria

**Keywords:** mortality salience, behavioral inhibition system, inhibition, worldview defense, approach-avoidance motivation, ethnocentrism

## Abstract

The hypothesis that people respond to reminders of mortality with closed-minded, ethnocentric attitudes has received extensive empirical support, largely from research in the Terror Management Theory (TMT) tradition. However, the basic motivational and neural processes that underlie this effect remain largely hypothetical. According to recent neuropsychological theorizing, mortality salience (MS) effects on cultural closed-mindedness may be mediated by activity in the behavioral inhibition system (BIS), which leads to passive avoidance and decreased approach motivation. This should be especially true for people motivated to avoid unfamiliar and potentially threatening stimuli as reflected in a high need for closure (NFC). In two studies involving moderated mediation analyses, people high on trait NFC responded to MS with increased BIS activity (as indicated by EEG and the line bisection task), which is characteristic of inhibited approach motivation. BIS activity, in turn, predicted a reluctance to explore foreign cultures (Study 1) and generalized ethnocentric attitudes (Study 2). In a third study, inhibition was induced directly and caused an increase in ethnocentrism for people high on NFC. Moreover, the effect of the inhibition manipulation × NFC interaction on ethnocentrism was explained by increases in BIS-related affect (i.e., anxious inhibition) at high NFC. To our knowledge, this research is the first to establish an empirical link between very basic, neurally-instantiated inhibitory processes and rather complex, higher-order manifestations of intergroup negativity in response to MS. Our findings contribute to a fuller understanding of the cultural worldview defense phenomenon by illuminating the motivational underpinnings of cultural closed-mindedness in the wake of existential threat.

*We encapsulate ourselves to avoid death. And life escapes us while we huddle within the defended fortress* (Becker, 1997, p. XIII).

## Introduction

According to cultural anthropologist Ernest Becker, humans are uniquely preoccupied as “terrified, death-avoiding animal[s]” (Becker, 1997, p. 99). Inspired by Becker's writings, terror management theory (TMT) proposes that humans avoid the threat of death on a symbolic level by identifying with something that outlasts their individual life, such as their nation or culture (Pyszczynski et al., 1997). In support of this proposition, numerous TMT studies have demonstrated that people respond to mortality salience (MS) with increased adherence to their cultural worldviews, which often manifests in avoidance and derogation of culturally different people, customs, and ideologies (Burke et al., 2010). Hence, this research reveals a fascinating irony inherent in human efforts at self-preservation: in order to manage death-related anxiety, people develop an aversion to the cultural “other,” thereby curtailing their opportunities for psychologically enriching experiences. In other words, an unfortunate consequence of “huddling in the defended fortress” is that people avoid sampling opportunities provided by getting to know other cultures and their members.

Although it is well-documented that MS can drive cultural closed-mindedness, little is known about the basic neural and motivational processes that underlie this effect. According to the process model of threat and defense (Jonas et al., 2014), MS-induced aversion to cultural otherness may be mediated by a very basic motivation to avoid punishment. In the present research, we test this assumption using neurobehavioral measures of the activity of the behavioral inhibition system (BIS) as measured by the line bisection task (LBT; Study 1) and frontal alpha asymmetry as measured by EEG (Study 2). We also directly manipulate the proposed inhibitory motivational mediator. Specifically, we investigate whether (a) MS activates inhibitory motivational processes, (b) inhibition mediates MS effects on cultural closed-mindedness, and (c) these effects are particularly strong for persons with a dispositional inclination to avoid unfamiliar and potentially threatening stimuli.

### Terror management and avoidance motivation

According to TMT, the awareness of one's inevitable demise entails the potential for paralyzing terror, which can be managed by defensive processes that are oriented toward symbolic self-preservation (Pyszczynski et al., 1997). Symbolic self-preservation is attained by identifying with entities that transcend one's individual death, such as cultural ingroups. Accordingly, there is ample evidence that MS increases cultural closed-mindedness in the form of more negative (positive) evaluations of the culturally unfamiliar (familiar; Burke et al., 2010).

Terror management theorists have generally assumed that these defenses are products of a basic motivation to avoid threatening cognitions (Pyszczynski et al., 1997, 2000, 2012). According to TMT, mortality threat is initially avoided by cognitive means, such as death-thought suppression or vulnerability denial (i.e., proximal defense; Pyszczynski et al., 1999). Second, after this active cognitive avoidance has subsided, subtle mechanisms of threat-avoidance are utilized to maintain defenses on a symbolic level (i.e., distal defense; Pyszczynski et al., 1999). These distal defenses often manifest in various forms of ethnocentric intergroup bias (e.g., avoidance and derogation of foreign people and worldviews; Burke et al., 2010). In other words, defensive responses to MS are assumed to be mediated by the motivation to avoid experiencing terror in the face of inescapable mortality (Greenberg et al., 2003). TMT argues that “when confronted with reminders of their mortality, people avoid the subjective experience of distress by increasing their commitment to the cultural worldview” (Greenberg et al., 1995b, p. 431). In sum, TMT proposes that threat leads people to shy away from cultural outgroups and cling to cultural ingroups in order to avoid the distress inherent in the existential insecurities aroused by reminders of the finitude of life.

### The general process model of threat and defense

Recent neuropsychological theorizing suggests that MS-induced discomfort with cultural otherness is rooted in a basic motivation to avoid negative/punishing outcomes (Jonas et al., 2014). Specifically, the process model of threat and defense (Jonas et al., 2014) states that MS activates the BIS, an avoidance motivational system that responds to distant or anticipated threat (such as one's eventual demise), which inhibits ongoing goal approach (McNaughton and Corr, 2004). The inhibition of goal approach functions to reorient the individual's attention so that threat can be resolved and viable goal pursuit resumed (cf. McGregor et al., 2009). This dynamic is accompanied by several attentional, affective, and motivational processes, including hypervigilance, anxious arousal, and the motivation to avoid threat by inhibiting behaviors that might put the individual at risk (passive avoidance; Gray and McNaughton, 2000; McNaughton and Corr, 2004; Corr et al., 2013a)^1^.

Importantly, upon detecting stimuli signaling the potential for conflict or punishment, the BIS further biases negativity, which entails a tendency to avoid everything that is unfamiliar and potentially threatening (McNaughton and Corr, 2004, 2014). Accordingly, passive threat-avoidance strategies include the inhibition of exploratory approach behavior in order to maintain distance from potentially threatening stimuli (e.g., here, potentially hostile outgroup members), especially in risky or unfamiliar contexts. From an evolutionary perspective, members of cultural outgroups represent unfamiliar and potentially threatening stimuli, since hostile strangers may have been among the most serious ancestral threats to survival (McEachron and Baer, 1982; see also Kenrick et al., 2010). Accordingly, we propose that reminders of mortality activate a basic threat-avoidance system—the BIS—that subsequently inhibits the motivation to engage with cultural outgroups. This proposed process is similar to the TMT account of MS sequelae, but it differs in one key way: people's defensive responses to MS are not merely *anticipatory* of a negative motivational-emotional state (in TMT, the *potential for anxiety*; Pyszczynski et al., 1999), but instead these defenses are driven by BIS-mediated processes, such as motivational inhibition and felt anxious uncertainty, that are aroused immediately following the contemplation of one's death.

Although increased ethnocentric thinking has been frequently found following MS (Burke et al., 2010), the mediating processes proposed by the general process model (Jonas et al., 2014) are to date largely hypothetical. Advancing on this empirical gap from the general process model is all the more important given what is arguably equivocal evidence for TMT's main cognitive mediator candidate—death-thought accessibility (Das et al., 2009; Golec de Zavala et al., 2012; Trafimow and Hughes, 2012; Agroskin and Jonas, 2013; Hart, 2014). This failure has spawned a lively debate in the existential threat literature about whether MS-induced ethnocentrism might be driven by specific needs to restore meaning (Proulx and Heine, 2010), certainty (van den Bos et al., 2005), security (Hart et al., 2005), a sense of coalition (Navarrete et al., 2004), or control (Fritsche et al., 2008). However, it has also been proposed that MS-induced defensiveness may be driven by rather basic processes such as approach motivational (McGregor et al., 2013) or unconsciously vigilant (Holbrook et al., 2011) responses to any kind of threat. By contrast, Jonas et al. (2014) have suggested that MS effects on ethnocentric cognitions and behaviors are attributable to BIS-generated passive avoidance of cultural novelty. The present research tests this novel notion.

### Individual differences in MS effects on cultural closed-mindedness

TMT research has demonstrated that individual differences in traits related to motivated avoidance of the unfamiliar are useful predictors of terror management processes. For instance, persons with high need for closure (NFC; Webster and Kruglanski, 1994), who strive for simple, unambiguous, and predictable environments, are accordingly reluctant to explore novel or ambiguous stimuli under threat, such as unfamiliar places (Vess et al., 2009)^2^. They also tend to adopt ethnocentric or xenophobic attitudes, suggesting that their discomfort with ambiguity and unfamiliarity biases their evaluations of cultural outgroups (Shah et al., 1998; Agroskin and Jonas, 2010, 2013). Accordingly, high NFC has been found to amplify MS effects on derogation of culturally different beliefs, such as differing religious worldviews (Juhl and Routledge, 2010). On a more general level, the unease with unfamiliar, unpredictable, and ambiguous stimuli following MS exhibited by people with high trait NFC can also manifest in heightened stereotypic thinking, as well as antipathy toward behaviorally inconsistent persons and even modern art (Schimel et al., 1999; Landau et al., 2004, 2006). Low NFC, conversely, is related to openness to novelty, uncertainty, and ambiguity, thus promoting increased interest in exploring unfamiliar stimuli following MS (Vess et al., 2009). In sum, high NFC appears to represent a disposition to avoidance-related cognition and behaviors, especially under threat.

This avoidance-motivational interpretation of NFC is bolstered by the fact that chronic BIS sensitivity and NFC are positively related (Corr et al., 2013b). This again suggests that high NFC may be indicative of the BIS-mediated negativity bias toward exaggerated threat perceptions and subsequent avoidance behaviors (McNaughton and Corr, 2004). The reluctance to engage with culturally-different stimuli following MS exhibited by high NFC people may reflect BIS-mediated inhibition of approach motivation under threat. On a more general level, Jonas et al. (2014) have proposed that BIS-related traits intensify and prolong BIS activity after threat. This is in line with neuroscientifically-informed theorizing regarding the hierarchical interplay between trait and state avoidance motivation (Elliot, 2006; Spielberg et al., 2013). In sum, people with high trait NFC are likely to show increased levels of BIS-mediated passive avoidance following MS, which may explain why NFC has been linked to many avoidance-related outcomes in previous TMT research.

### The role of BIS activity in mortality salience effects

Although the hypothesis that MS effects on ethnocentric thinking are mediated by BIS activity has not yet been explicitly tested yet, there is some evidence suggesting an association between MS and BIS-related processes. For instance, people prefer familiar over unfamiliar products after experiencing death anxiety, suggesting a basic tendency toward novelty-avoidance (Huang and Wyer, 2015). MS also causes people to avoid stimuli that remind them of their embodied, temporal existence (Goldenberg et al., 2001; Cox et al., 2007), which suggests that MS inclines an avoidance of information that would further highlight a threat. Finally, people are even reluctant to look into mirrors and engage in self-focused writing under MS, possibly because heightened self-awareness exacerbates distressing vulnerability concerns after contemplating one's own death (Arndt et al., 1998).

In line with the process model of threat and defense (Jonas et al., 2014), these avoidance reactions are particularly likely to occur for individuals with anxious personalities. For example, MS has been found to aggravate anxious and avoidant behaviors among people especially prone to phobic or compulsive behaviors (Strachan et al., 2007), such as people high on BIS and trait anxiety (Smits and Boeck, 2006; Bijttebier et al., 2009; Erdle and Rushton, 2010). Furthermore, stimuli related to physicality and creatureliness are particularly problematic for persons high in neuroticism after MS (Goldenberg et al., 2000, 2006a,b, 2008). Finally, research from diverse labs demonstrates novelty-avoidant, risk-averse behaviors under conditions of threat and anxiety (e.g., Maner and Schmidt, 2006; Maner et al., 2007; Mortensen et al., 2010; Litt et al., 2011; Clark et al., 2012; see Kenrick et al., 2010 for review), and these reactions appear to be especially likely for people high on BIS-related traits (Landau and Greenberg, 2006; Cavallo et al., 2009; Routledge et al., 2010). Taken together, these results are consistent with the notion that BIS-sensitive people are particularly prone to passive-avoidant behaviors in the wake of threat (Jonas et al., 2014).

### Measuring BIS activity

In neural terms, BIS activity has been linked to relative right-hemispheric brain activity, as reflected in prefrontal EEG alpha asymmetry. Specifically, dispositional BIS sensitivity is associated with stronger relative right-frontal brain activation, whereas BAS sensitivity is robustly related to greater left-frontal activation (Sutton and Davidson, 1997; Shackman et al., 2009). There is also evidence linking frontal asymmetric activity to prevention/promotion regulatory focus, which is conceptually related to BIS/BAS activity, respectively (Amodio et al., 2004). Importantly, relative frontal EEG activity is computed using difference scores between right- and left-frontal electrodes, implying that increased BIS-related right-frontal asymmetry is functionally equivalent to reduced BAS-related left-frontal asymmetry and vice-versa (e.g., Sutton and Davidson, 1997). This is also consistent with an inverse interrelationship between the activity of both hemispheres (e.g., Amodio et al., 2004) and the joint subsystems hypothesis of BIS/BAS activity in reinforcement sensitivity theory (Corr, 2004), which states that the two systems mutually inhibit each other. Thus, BIS-mediated inhibition of approach motivation is equivalently reflected in reduced left-frontal asymmetry as well as increased right-frontal asymmetry in the context of EEG asymmetry scores (see also Coan and Allen, 2004).

Moreover, right-frontal EEG asymmetry has been found to contribute to numerous neural and behavioral indicators of anxious or inhibitory responding. For example, relative right-frontal activity is positively associated with state anxiety (Davidson et al., 2000; Harmon-Jones et al., 2009) and hypervigilance to threat (Pérez-Edgar et al., 2013; Grimshaw et al., 2014). It also predicts the amplitude of the error-related negativity (Nash et al., 2012), which is linked to BIS sensitivity (Boksem et al., 2006, 2008). Importantly, it has also been demonstrated that right-frontal asymmetry is specifically related to the anxious inhibitory state of *passive* avoidance, whereas left-frontal asymmetry has been linked to the fearful state of *active* avoidance (Wacker et al., 2008; see also Perkins et al., 2007 for behavioral evidence on the fear/anxiety distinction). Overall, there is a substantial body of evidence to support the notion that right-frontal EEG asymmetry is associated with numerous neural and behavioral indicators of anxious or inhibitory responding (for more evidence, see Thibodeau et al., 2006; Wacker et al., 2010; McNaughton et al., 2013). Convergent evidence from functional magnetic resonance imaging (fMRI) has also demonstrated a link between right-frontal brain activity and anxiety (e.g., Dalton et al., 2005; Engels et al., 2010) and inhibition (see Aron et al., 2004 for review).

Nash et al. (2010) demonstrated that BIS/BAS activity can also be investigated using the line bisection task (LBT) as a perceptual measure of relative prefrontal hemisphericity. The LBT gauges BIS/BAS activity (or left-frontal asymmetry) by determining the extent to which people's perceptions of the midpoints of horizontal lines are biased to the right visual field, mirroring neural activity in the contralateral hemisphere (Nash et al., 2010). Accordingly, reduced rightward line bisection bias (i.e., reduced left or increased right hemisphericity) has been associated with BIS-related phenomena, such as anxious-avoidant arousal (Friedman and Förster, 2005), feelings of passivity/inhibition, and powerlessness (Drake and Myers, 2006; Wilkinson et al., 2010), as well as reduced risk taking, optimism, and goal pursuit (Drake and Ulrich, 1992; Nash et al., 2011). Notably, line bisection bias is specifically related to frontal (F7/F8) but not central, parietal, temporal, or occipital asymmetry, suggesting that the LBT represents a neurobehavioral marker specific to frontal alpha asymmetry (Nash et al., 2010). In sum, the LBT has often been used to measure motivational tendencies (see Vallortigara and Rogers, 2005 for a review of motivationally-relevant perceptual asymmetries), and has been validated as a neurobehavioral measure of asymmetric frontal brain activation (Nash et al., 2010).

### The present research

In the present research, we examined the hypotheses that (a) MS causes BIS activity, evidenced by increased by greater right-hemispheric frontal asymmetry, (b) BIS activity mediates MS effects on cultural closed-mindedness, and (c) these effects are pronounced among persons with high NFC. Stated differently, we tested the moderated mediational hypothesis that MS effects on cultural closed-mindedness are mediated by BIS activity and are particularly strong for persons with high NFC. These hypotheses were examined by inducing MS (vs. a control topic), measuring NFC, measuring frontal brain asymmetry via the LBT (Study 1) and EEG (Study 2), and measuring cultural closed-mindedness in the form of reluctance to engage with cultural novelty (Study 1) and general ethnocentrism (Study 2). Moreover, in order to substantiate our claim that inhibition is the mediator, we directly manipulated the proposed mediator—inhibition—in Study 3 to test whether inhibition evokes ethnocentric thinking for high NFC persons (cf. Spencer et al., 2005).

## Study 1

Study 1 was designed to test whether (a) persons with high NFC respond with increased BIS activity (as assessed by LBT-assessed right-frontal activity) to MS, and (b) their increased BIS activity mediates their increased unwillingness to explore foreign cultures.

### Method

#### Participants and design

Seventy-seven students at the University of Salzburg participated in this paper-pencil study, which took place in class, prior to a lecture^3^. Three participants failed to follow instructions and were dropped prior to analyses, leaving a final sample of 74 participants (54 females and 20 males). Their mean age was 22.0 years (*SD* = 1.8; range: 19–31). Participant nationalities were 54 German, 18 Austrian, and 2 other. Participants were randomly assigned to one of two conditions, MS (*N* = 35) or TV salience (*N* = 39), in a between-subjects design. The study was described as an investigation of personality and visual perception. The study was approved by the ethics committee of the University of Salzburg. All participants signed informed consent, and could withdraw participation at any point, although no participant made use of this option.

### Procedure and materials

#### Need for closure

Following instructions and the baseline LBT measure (described in more detail below), the NFC scale was presented. We measured NFC with three items that were taken from the German version of the NFC scale (Webster and Kruglanski, 1994; Schlink and Walther, 2007). The items were selected *a priori* based on two criteria: First, representativeness of the whole NFC scale, that is, corrected item-total correlations presented by Schlink and Walther (2007). Second, the items that best capture the willingness to approach/avoid novel and unpredictable stimuli should be of particular relevance (Green and Campbell, 2000), contrary to decisiveness, which is also encompassed by the NFC scale (see also Neuberg et al., 1997 for a critique of the full NFC scale's conceptual imprecision). One item was dropped prior to analyses because its corrected item-total correlation suggested it was unrelated with the other two items in the current sample (*r*_*it*_ = 0.00). The two remaining items were: “I prefer familiar things over unfamiliar and unpredictable ones” and “I don't like unpredictable situations” (interrelation: *r* = 0.53, *p* < 0.001). A pilot study revealed this short NFC scale to correlate strongly with the conceptually related personal need for structure scale by Neuberg and Newsom (1993), *r* = 0.72, *p* < 0.001, *N* = 217. Responses were given on a 1 (*almost never*) to 6 (*almost always*) scale, and averaged to create a single composite score (*M* = 3.93, *SD* = 0.85).

#### Manipulation

After a few further personality items, included to bolster the cover story, we presented the classic MS manipulation, asking participants two open-ended questions about what will happen to them physically when they die and after they are dead, and which emotions are caused by thinking about their own death. Participants in the control condition were asked parallel questions about watching TV.

#### Line bisection task (inhibition of approach motivation)

Following a free-thought delay of a few minutes, the LBT was presented for a second time (the first LBT was presented right at the beginning of the study to obtain a baseline LBT measure). In each measure, participants were instructed to mark the perceived center point of eight horizontal lines that ranged from 4 to 6 (102 to 152 mm) in length with their centers offset from each other (see also Drake and Myers, 2006, who used the same LBT). Estimation errors to the left reflect an overnoticing of the left visual field characteristic of relative right cerebral hemisphericity (Jewell and McCourt, 2000). Thus, we computed the index of relative right cerebral hemisphericity by subtracting each participant's number of right-of-center ticks from left-of-center ticks (see McGregor et al., 2010, Study 1). In line with prior research, both LBT measures were correlated, *r* = 0.67, *p* < 0.001 (McGregor et al., 2010), and participants exhibited a leftward bisection bias on average (LBT 1: *M* = 1.92, *SD* = 4.12; LBT 2: *M* = 1.10, *SD* = 3.80; Jewell and McCourt, 2000).

#### Avoidance of cultural novelty

Passive avoidance of cultural novelty was measured with eight researcher-generated items designed to measure interest in exploring culturally foreign stimuli (e.g., people, information). Based on the conceptual view that exploration constitutes approach behavior toward novel stimuli (Green and Campbell, 2000), and evidence implicating BIS activity in reduced exploratory approach behaviors (Green and Campbell, 2000; Elliot and Reis, 2003; Kashdan et al., 2004), we recoded our cultural exploration items to obtain a measure for passive avoidance of cultural novelty. Example items include “I would like to get to know people from foreign cultures” and “I am curious to learn how people live in foreign cultures^4^.” Participants were asked to indicate the extent to which the items describe them at the present moment. Responses were given on a 1 (*very little*) to 5 (*very much*) scale, and averaged to create a single composite score (8 items; *M* = 2.40, *SD* = 0.80, α = 0.90).

After that, we included some newly created personality items that were unrelated to an interest in exploring culturally foreign stimuli, a newly created cross-cultural conflict resolution task that included a manipulation, and a criterion variable for explorative reasons. Finally, we collected some demographic data and measured participants' handedness (Oldfield, 1971).

### Results

#### Threat × NFC on cultural novelty avoidance and avoidance motivation

**Figure 1:**
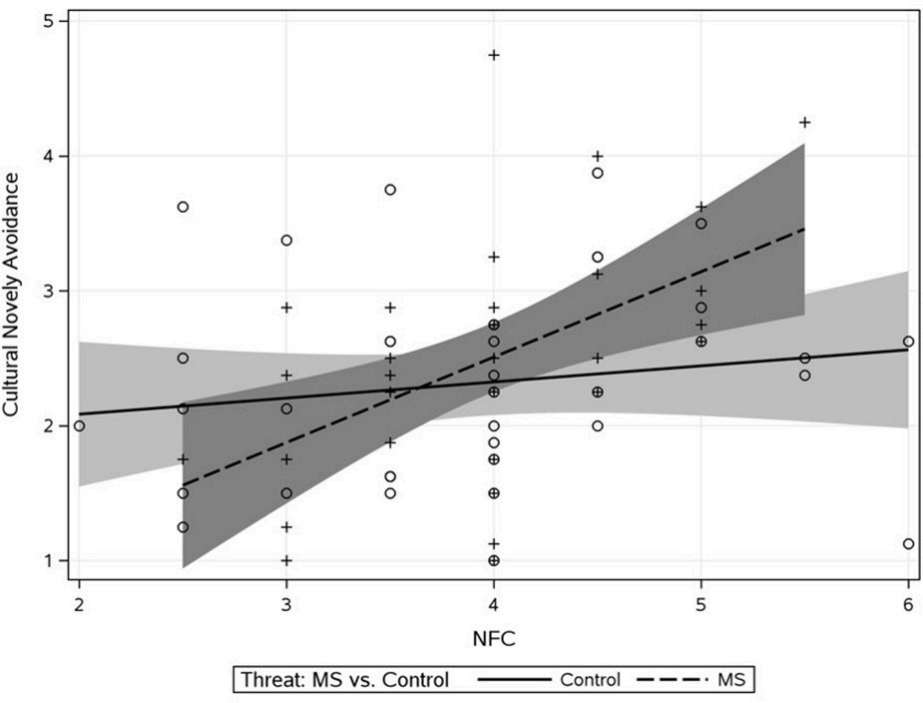
**Study 1 effect of threat × NFC interaction on cultural novelty avoidance**. Shaded regions represent 95% confidence bands on the regression lines.

As an initial step we sought to confirm that MS heightens avoidance of cultural otherness especially at high NFC. For this, effect-coded threat (with zero as the non-threat and one as the threat condition), NFC, and the threat × NFC term were entered as predictors of cultural avoidance. Results are displayed in Table 1. Both the main effects of threat and NFC were significant. These main effects were qualified by the predicted interaction (Δ*R*^2^ = 0.06) such that avoidance of cultural novelty was especially high under MS vs. control for people high (+1 *SD*) on NFC [*b* = 0.59, *SE* = 0.26, *t*_(70)_ = 2.28, *p* = 0.025, see Figure 1]. There was no effect of threat at low NFC (−1 *SD*), *b* = −0.26, *SE* = 0.26, *t*_(70)_ = 1.09, *p* = 0.277.

**Table 1:** **Regression statistics for the prediction of cultural novelty avoidance, Study 1**.

	***b***	***SE***	**95% CI**		***t***	***p***	**β**	**Partial *r*^2^**
Threat	0.07	0.09	−0.10	0.25	0.85	0.396	0.09	0.01
NFC	0.38	0.11	0.15	0.60	3.34	0.001	0.40	0.14
Threat × NFC	0.26	0.11	0.03	0.48	2.28	0.026	0.27	0.07

*N = 74. Model R^2^ = 0.16*.

**Figure 2:**
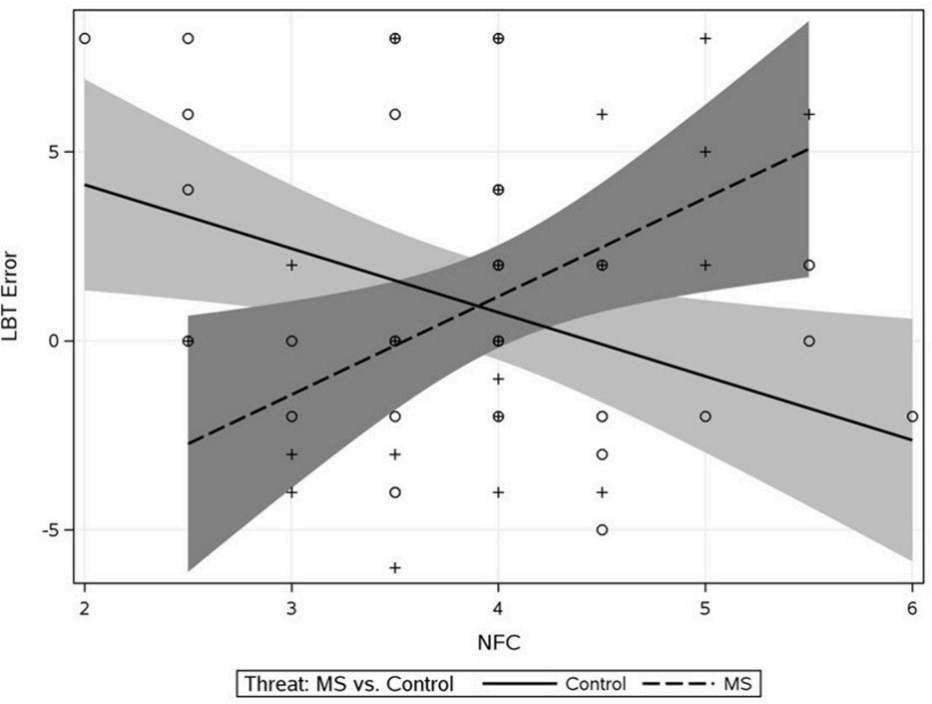
**Study 1 effect of threat × NFC interaction on LBT bisection errors**. Shaded regions represent 95% confidence bands on the regression lines. Positive values indicate leftward bisection bias.

Next, we tested our key hypothesis that MS causes BIS activity—as indicated by a shift in leftward bisection bias—for persons with high NFC (the mediator model). In line with prior research using the LBT and to control for hemispheric differences arising from handedness (e.g., Drake and Myers, 2006), we excluded 16 left-handed individuals from all analyses involving the LBT (Oldfield, 1971). Furthermore, the LBT baseline measure was entered as a predictor in all analyses including the LBT; thus, effects on LBT can be considered demonstrations of residual change. Baseline LBT, effect-coded threat, NFC, and the threat × NFC interaction were entered as predictors of post-threat LBT. As displayed in Table 2, there were no main effects, and the threat × NFC interaction was significant (Δ*R*^2^ = 0.060). Avoidance of cultural novelty was especially high under MS vs. control for people high on NFC, though this effect did not emerge until very high values of NFC (~+2 *SD* or the 95% of our observed distribution according to the Johnson-Neyman technique, 1936). There was an unpredicted effect of threat at low NFC (−1 *SD*), *b* = −2.42, *SE* = 1.11, *t*_(53)_ = 2.17, *p* = 0.034. Figure 2 shows that the effect of NFC on bisection bias under MS reversed from control such that NFC predicted more avoidance under MS.

**Table 2:** **Regression statistics for the prediction of post-threat LBT bisection error**.

	***b***	***SE***	**95% CI**		***t***	***p***	**β**	**Partial *r*^2^**
Threat	−0.08	0.36	−0.81	0.65	−0.22	0.827	−0.02	0.00
NFC	−0.06	0.50	−1.06	0.95	−0.12	0.907	−0.01	0.01
Threat × NFC	1.32	0.51	0.29	2.34	2.57	0.013	0.27	0.15
Baseline LBT	0.56	0.09	0.38	0.74	6.29	0.001	0.61	0.36

*N = 58. Model R^2^ = 0.52*.

#### Test of moderated mediation

**Figure 3:**
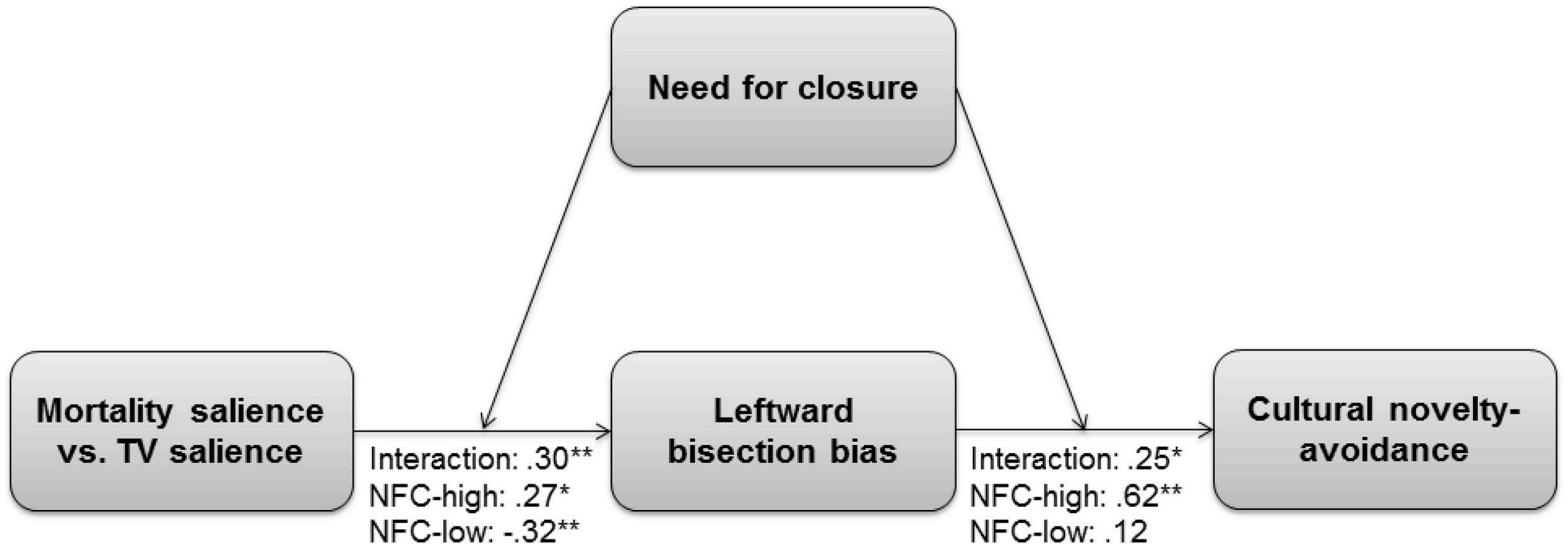
**Moderated mediation in Study 1**. Mortality salience is coded as 1 and TV salience as 0. NFC-high = effects of the focal predictors (i.e., MS and leftward bias) under conditions of high need for closure (*SD* = 1). NFC-low = effects of the focal predictors (i.e., MS and leftward bias) under conditions of low need for closure (*SD* = −1). Standardized regression coefficients (β) are indicated. ^***^p ≤ 0.001, ^**^p ≤ 0.01, ^*^p ≤ 0.05.

Next, we tested whether BIS activity transmits or explains the effect of the interaction of MS and NFC to cultural avoidance. Because NFC is linked to both sensitivity to threat and motivated closed-mindedness under threat, it was allowed to moderate both the path from the threat to the mediator, as well as from the mediator to the dependent variable (i.e., model 58; Hayes, 2013). Results of this analysis are displayed in Figure 3. In sum, the effect of post-threat BIS activity explained the effect of threat × NFC on cultural novelty avoidance, especially at high levels of BIS and NFC.

### Brief discussion

These results provide the first evidence for the notion that (a) MS leads to increases in BIS activity, (b) BIS activity mediates MS effects on aversion to cultural novelty, and (c) these effects are particular to people high on NFC. Thus, the findings demonstrate that a basic, passive-avoidance motivational process underlies higher-order manifestations of behavioral inhibition, such as the reluctance to explore unfamiliar cultures. This dynamic was only present for high NFC individuals, suggesting that they are particularly inclined to avoid the terror of death through motivated closed-mindedness toward cultural otherness.

One limitation of the current study was that our cultural novelty-avoidance measure had not been used prior to the study, so its construct validity was questionable. We thus conducted a follow-up study to explore the relationship between this measure and a frequent operationalization of cultural worldview defense. We found that the cultural novelty-avoidance measure used in Study 1 correlated with a measure of general ethnocentrism, *r*_(30)_ = 0.37, *p* = 0.037. This ethnocentrism measure has been used as a worldview defense measure in prior TMT research (Agroskin and Jonas, 2013), and found to be related to NFC (Agroskin and Jonas, 2010). Thus, our cultural novelty-avoidance items may at least partly tap into those destructive aspects of ethnocentric intergroup bias that are characteristic of cultural worldview defense.

Study 1 provided strong initial support for the proposition that MS activates the BIS particularly in high NFC people, which explains their post-threat closed-mindedness. However, we used a fairly indirect measure of frontal asymmetry measured via the LBT. In Study 2, we turned to a more direct measure of frontal asymmetry available via EEG. Furthermore, Study 1 employed a short NFC scale instead of the full measure. Thus, Study 2 provides an opportunity for replication and extension of the findings of Study 1.

## Study 2

Building on Study 1, Study 2 employed a poem-based MS manipulation (Agroskin and Jonas, 2013), the full NFC scale (Schlink and Walther, 2007), and frontal EEG alpha asymmetry instead of the LBT as a more direct measure of BIS-related neural activity. We also used the general ethnocentrism measure from the follow-up study instead of cultural novelty-avoidance to tap into the destructive aspects of cultural closed-mindedness that often characterize worldview-defensive negativity toward cultural outgroups. Summing up, Study 2 was designed to provide multimethodological and conceptual replication for the findings of Study 1.

### Method

#### Participants and design

The sample consisted of 33 participants (23 female) from the University of Salzburg without any reported history of neurological disorders or prior head trauma^5^. Their mean age was 22.1 years (*SD* = 3.0; range: 18–32)^6^. Twenty-two participants held German and eleven Austrian nationalities. Participants were randomly assigned to one of two conditions, MS (*N* = 16) or TV salience (*N* = 17), in a between-subjects design^7^. All participants gave written informed consent to participate in the study, which ostensibly investigated topics associated with literature and personality. They were rewarded with course credits. The study was approved by the ethics committee of the University of Salzburg. All participants signed informed consent, and could withdraw participation at any point, although no participant made use of this option.

### Procedure and materials

#### Need for closure

Prior to coming to the lab for the EEG measurement, participants were emailed and asked to fill out a few online questionnaires, including the whole NFC scale (Schlink and Walther, 2007) whose short form was used in Study 1^8^. Responses were given on a 1 (*totally disagree*) to 6 (*totally agree*) scale, and averaged to create a single composite score (14 items; *M* = 3.23, *SD* = 0.70, α = 0.82)^9^.

#### Manipulation

Upon arriving at the lab, participants were asked to provide some demographic data. After that, they underwent a 90-s baseline EEG recording (see below for more detail). Then, they were presented a short poem related to death or a neutral control topic (weather). The death-related poem has been used as a MS manipulation before (Agroskin and Jonas, 2013, Study 2). Literally translated from German, it reads “The hardest and cruelest power, which man has to accept, is death.” The weather-related poem reads “Look to the sky, clouds are passing by. Some are raining, others are not. Some are flashing, others are not.” Participants were asked to read the poem and describe how they interpret it, what they associate with it (e.g., images, sounds, smells, and moods), whether they have experienced anything in their own lives that showed them that the poem was true, and whether their own existence had certain aspects reflecting the veracity of the poem. These questions were used to help ensure a deep and thorough processing of the poems and their meaning.

#### EEG (inhibition of approach motivation)

Right after the manipulation, participants underwent another 90-s EEG recording in order to assess post-threat avoidance motivation as a function of MS. During all EEG recordings participants were asked to fixate a small black cross in the middle of the screen. After this EEG measurement, participants filled out the 20-item Positive and Negative Affect Schedule (PANAS; Watson et al., 1988). Then, participants underwent one more 90-s EEG recording.

#### Ethnocentrism

Following a delay of a few minutes (involving questions about which TV programs participants would like to watch and their reactions to two press reports about crimes^10^), which is necessary to obtain MS effects following explicit MS inductions (Pyszczynski et al., 1999), general ethnocentrism was measured using a short form of the ethnocentrism scale by Bizumic et al. (2009). This short form has been previously used by Agroskin and Jonas (2013) as a measure of cultural worldview defense, and was found to be associated with NFC, right-wing authoritarianism, avoidance of empathy, and anti-immigration attitudes (Agroskin and Jonas, 2010). Sample items are “In most circumstances it is right and natural to favor members from one's own cultural or ethnic group over strangers or foreigners” and “We need to do what's best for our own people, and stop worrying so much about what the effect might be on other peoples.” Responses were given on a 1 (*totally disagree*) to 6 (*totally agree*) scale, and averaged to create a single composite score (4 items; *M* = 2.10, *SD* = 0.91, α = 0.71)^11^.

Then, for exploratory reasons, participants underwent another 90 s EEG recording trial and completed some questions about their attitudes toward the European currency. Finally, they indicated their nationality and were probed for suspicion. Our focal BIS-related hypotheses were not correctly guessed by any participant^12^.

#### EEG apparatus and data reduction

We used a 14-channel Emotiv EEG neuroheadset (Emotiv Systems Inc., San Francisco, CA, USA), recording data with Emotiv TestBench software at a sampling rate of 128 Hz. EEG data was recorded at sites AF3, AF4, F3, F4, F7, F8, FC5, FC6, P7, T7, T8, P8, O1, and O2 in accordance with the 10–20 International System. Two mastoid electrodes served as online reference. The Emotiv EEG neuroheadset employs gold-plated contact-grade hardened copper electrodes with saline-moistened felt pads to record EEG. Importantly, the Emotiv EEG device has been found to record similar suppression of alpha power (8–13 Hz) in the Left Precentral AAL (Automated Anatomical Labeling) region in response to imagined right-finger tapping trials as compared to a standard Biosemi Active-II 64-channel device (see also Stopczynski et al., 2014a,b). This suggests that the Emotiv device is suitable for investigating neural processes associated with regional changes in alpha power. Further, validation studies determined that when used no longer than about 60 min per recording session, the ERP signal quality produced by this device is comparable to (or only slightly lower than) the signal quality provided by traditional EEG systems (Badcock et al., 2013; Mayaud et al., 2013). Consequently, Emotiv EEG technology has recently been increasingly used for cognitive neuroscience and brain-computer interface (BCI) applications (Bobrov et al., 2011; Debener et al., 2012; Louwerse and Hutchinson, 2012; Choi and Jo, 2013; Khushaba et al., 2013; O'Regan et al., 2013; O'Regan and Marnane, 2013; De Vos et al., 2014; Vourvopoulos and Liarokapis, 2014; Aspinall et al., 2015; Steinhubl et al., 2015).

Using BrainVision Analyzer 2.0 (Brain Products, Gilching, Germany), EEG data were filtered with an IIR filter (high-pass cutoff: 0.1 Hz, Slope: 24 db/Oct; low-pass cutoff: 30 Hz, Slope: 24 db/Oct). Next, the three 90-s EEG recording epochs were extracted from the continuous data and were further segmented into 2s segments (1.5s overlap). Epochs were included in condition averages when a difference between two values in a moving 200 ms interval did not exceed 100 microvolt, or when the signal was below −100 or above +100 microvolt. The 2s segments were then Fourier transformed (Fast Fourier Transform, 10% Hamming Window, frequency resolution 0.5 Hz) and the resulting power spectra were averaged. Individual frontal asymmetry scores (μV^2^) for each epoch were calculated (log F7–log F8) in the alpha band (8–13 Hz) and exported for statistical analysis. Positive values index right-frontal hemisphericity indicative of avoidance motivation. Analyses focused on lateral frontal asymmetry (F7–F8) because these electrodes have been specifically linked to bisection bias in the LBT (Nash et al., 2010).

### Results

#### Preliminary analyses

Frontal asymmetry scores (L – R alpha) for the proximal and distal recordings were baseline adjusted for each participant so that they reflected changes in asymmetry, following past research on state frontal alpha asymmetry (e.g., Allen et al., 2001; Ravaja et al., 2013). The resultant asymmetry scores for the proximal and distal recordings were highly correlated, *r* = 0.63, *p* < 0.001. Given that Jonas et al. (2014) assume anxious persons to show prolonged BIS activity following threat, and NFC is known to affect proximal and distal MS effects in a similar way (Juhl and Routledge, 2010), we merged the proximal and distal asymmetry scores.

### Primary analyses

#### Analytical strategy

As in Study 1, the hypotheses were tested in several steps using moderated mediational analyses (Hayes, 2013) performed using Mplus 7 (Muthén and Muthén, 2012). The zero-order correlations are not explicitly reported because they were virtually identical (i.e., same directions and similar magnitudes and significance levels) with the respective main effects reported below^13^.

#### MS × NFC effect on EEG-inhibition

**Figure 4:**
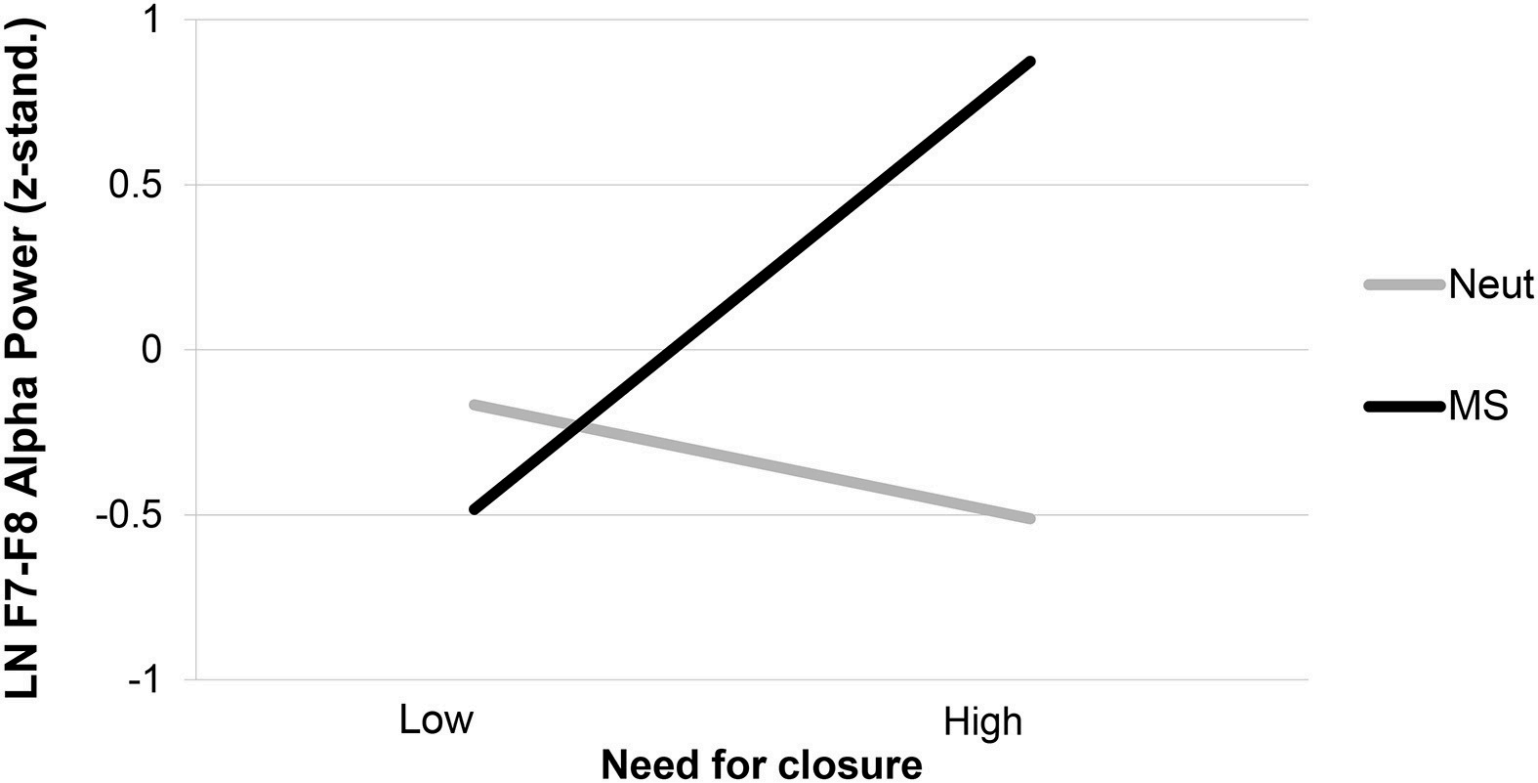
**Inhibition of approach motivation measured by LN F7-F8 alpha power (μV^2^) as a function of need for closure (NFC) and mortality salience (MS)**. Neut, neutral control topic.

First, we tested whether individuals with high NFC showed higher right-frontal (or reduced left-frontal) asymmetry following MS (coded as MS = 1, weather = 0) by regressing the post-threat asymmetry score onto pre-threat asymmetry, threat, NFC, and the threat × NFC interaction. There was no effect of NFC in the control condition, *b* = −0.05, *SE* = 0.06, *t*_(28)_ = 0.78, *p* = 0.443 and a significant main effect of threat such that avoidance was higher under MS than control, *b* = 0.69, *SE* = 0.31, *t*_(28)_ = 2.23, *p* = 0.025. This effect was qualified by a significant interaction between the manipulation and NFC, *b* = 0.24, *SE* = 0.09, β = 0.43, *t*_(28)_ = 2.56, *p* = 0.01, Δ*R*^2^ = 0.16 (see Figure 4). Simple effects analyses revealed that high NFC individuals (*SD* = 1) showed the predicted increase in right-frontal asymmetry following MS, *b* = 0.27, *SE* = 0.10, β = 0.69, *t*_(28)_ = 2.64, *p* < 0.01, whereas this was not true for low NFC persons (*SD* = −1), *b* = −0.06, *SE* = 0.10, β = −0.16, *t*_(28)_ = −0.65, *p* = 0.52. Thus, our hypotheses were corroborated: MS increased BIS-based inhibition of approach motivation but only for high NFC persons.

#### EEG-inhibition effect on ethnocentrism

Right-frontal asymmetry was entered as a predictor in addition to the manipulation and NFC. The interaction between the manipulation and NFC (i.e., direct interactive effect) was fixed to zero because we assumed a complete mediation model (Shrout and Bolger, 2002). As expected, right-frontal asymmetry predicted ethnocentrism, *b* = 2.49, *SE* = 0.69, β = 0.54, *t*_(29)_ = 3.59, *p* < 0.001. No other relationships attained significance (*p*s > 0.34). In contrast to Study 1, adding the interaction between right-frontal asymmetry and NFC did not reveal moderation by NFC, *t*_(27)_ = −0.62, *p* = 0.53. Thus, BIS activity affected ethnocentrism regardless of NFC levels. We thus fixed the effect of NFC to zero in accordance with Hayes (2013, Model 7) for the moderated mediational analyses. Summing up, the more participants had right-frontal asymmetry, the more they adopted ethnocentric attitudes. Our hypotheses were therefore supported.

#### Bootstrap estimation of the indirect effects

**Figure 5:**
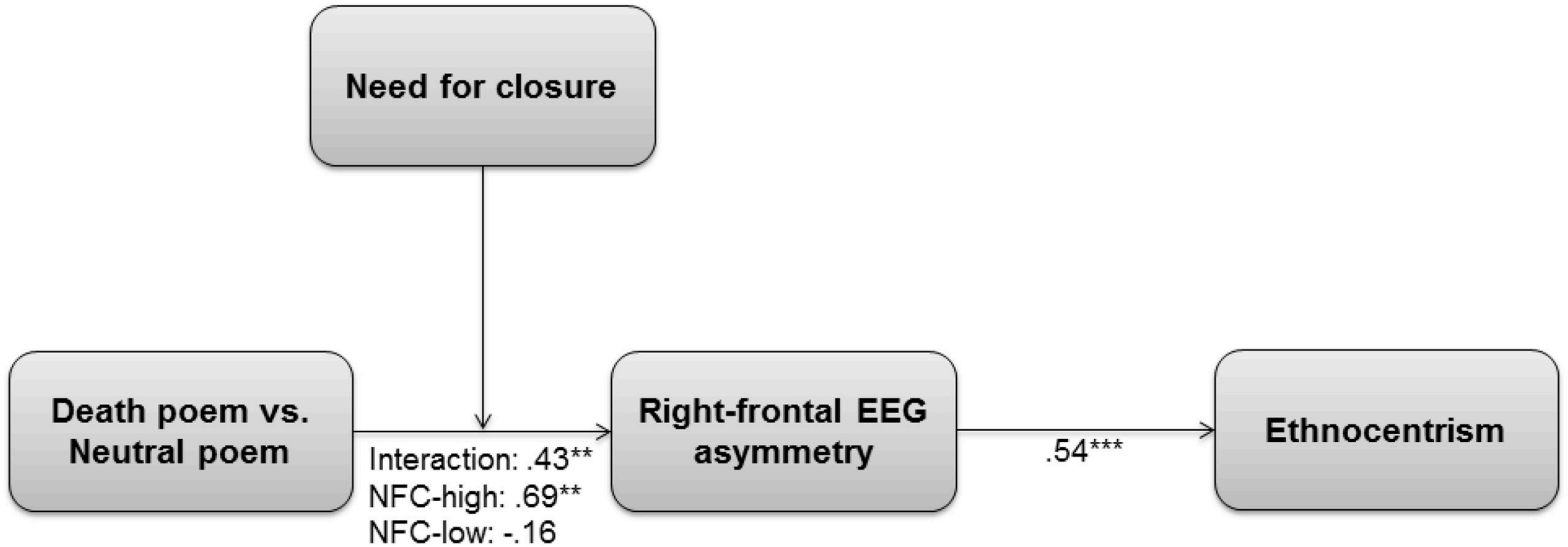
**Moderated mediation in Study 2**. The death-related poem was coded as 1 and the neutral poem as 0. NFC-high, MS effects under conditions of high need for closure (*SD* = 1). NFC-low, MS effects under conditions of low need for closure (*SD* = −1). Standardized regression coefficients (β) are indicated. ^***^p ≤ 0.001, ^**^p ≤ 0.01, ^*^p ≤ 0.05.

As per bias-corrected bootstrap procedure using 75000 bootstrap samples (Hayes, 2013, Model 7, see Figure 5), MS significantly increased ethnocentrism through right-frontal asymmetry for high NFC persons, point estimate (*b*) = 0.67, 95% CI [0.02, 1.43]. In contrast, there was no conditional indirect effect for low NFC persons, *b* = −0.15, 95% CI [−0.84, 0.33].

Finally, the overall model test was consistent with our hypothesis of complete mediation (Hayes, 2013, Model 7), $\chi_{\left(2\right)}^{2}$ = 0.65, *p* = 0.72, CFI = 1.00. Thus, our key mediational hypothesis for high NFC individuals was supported: MS amplified ethnocentric thinking via the BIS-based inhibition of approach motivation^14^.

### Discussion

These results replicate and expand the findings from Study 1 in several important ways. First, they provide a more direct measure of right-frontal asymmetric activity in the prefrontal cortex via EEG. Second, they involve another operationalization of motivated closed-mindedness in the cultural domain—generalized ethnocentric attitudes—which suggests that a similar process may underlie various forms of worldview defense. All in all, the findings are clearly supportive of the view that high NFC persons' approach motivation is strongly inhibited following mortality reminders, which underlies their inclination to engage in ethnocentric thinking.

In Study 3, we aimed at conceptually replicating the effect of BIS-related inhibition on ethnocentrism by directly manipulating inhibition. Further, this test provides another means of testing for mediation by manipulating the proposed mediator (Spencer et al., 2005).

## Study 3

In Study 3, we employed an experimental approach to testing mediation by manipulating the assumed mediator—inhibition. Building on research on disinhibition by van den Bos et al. (2009), we developed a novel manipulation of inhibition to test whether bringing people into a state of inhibition produces the same ethnocentric bias as threat and BIS activity. Thus, we measured NFC, induced inhibition, and measured ethnocentrism with the same instrument as in Study 2. In addition, we included a BIS affect measure as a manipulation check. We expected inhibition to promote ethnocentric thinking for persons with high NFC.

### Method

#### Participants and design

Seventy-six students at the University of Salzburg participated in this paper-pencil study, which took place in class, after a lecture^15^. Participants were 58 females, 15 males, and 3 undisclosed. Their mean age was 21.6 years (*SD* = 2.0; range: 19–30). Fifty-five participants held German, 17 Austrian, and 1 another nationality (3 undisclosed). Participants were randomly assigned to one of two conditions, inhibition (*N* = 36), or control (*N* = 40), in a between-subjects design. The study was described as an investigation of participants' personalities. The study was approved by the ethics committee of the University of Salzburg. All participants signed informed consent, and could withdraw participation at any point, although no participant made use of this option.

### Procedure and materials

#### Need for closure

Because the two-item NFC measure used in Study 1 was shown to be sensitive to MS, we measured NFC with same two items (*M* = 3.77, *SD* = 0.85; *r* = 0.44, *p* < 0.001).

#### Manipulation

After a few further personality items, included to bolster the cover story, we presented a novel manipulation of inhibition. Building on the disinhibition manipulation from van den Bos et al. (2009), we asked participants three open-ended questions about behaving with inhibitions: “Please briefly describe a situation out of your own life in which you felt and behaved in an inhibited way like you typically do.” In the next two questions, we asked participants how they behaved and felt in this situation. In the control condition, we posed the same three questions referring to their behaving in a normal way (cf. van den Bos et al., 2009): “Please briefly describe a situation out of your own life in which you felt and behaved in a normal way like you typically do.” The other two questions referred to participants' behavior and feelings again.

#### Ethnocentrism

Ethnocentrism was measured in the same way as in Study 2 (4 items; *M* = 1.94, *SD* = 0.77, α = 0.60)^16^.

#### BIS affect

Following Schumann et al. (2014), we included a retrospective manipulation check asking about how participants felt when responding to the inhibition/control condition-related questions. We presented five items measuring BIS-related affect, including four adjectives from the German translation of the Carver and White (1994) BIS sensitivity scale (Strobel et al., 2001), namely *nervous* (German: *nervös*), *anxious* (ä*ngstlich*), *worked up* (*unruhig*), and *worried* (*besorgt*), along with the face-valid adjective *inhibited* (*gehemmt*; five items; *M* = 2.32, *SD* = 1.45, α = 0.94). Participants responded on a scale ranging from 1 (*not at all*) to 5 (*extremely*). Finally, participants completed the PANAS (Watson et al., 1988) and some demographic questions^17^.

### Results

#### Inhibition × NFC on ethnocentrism

**Figure 6:**
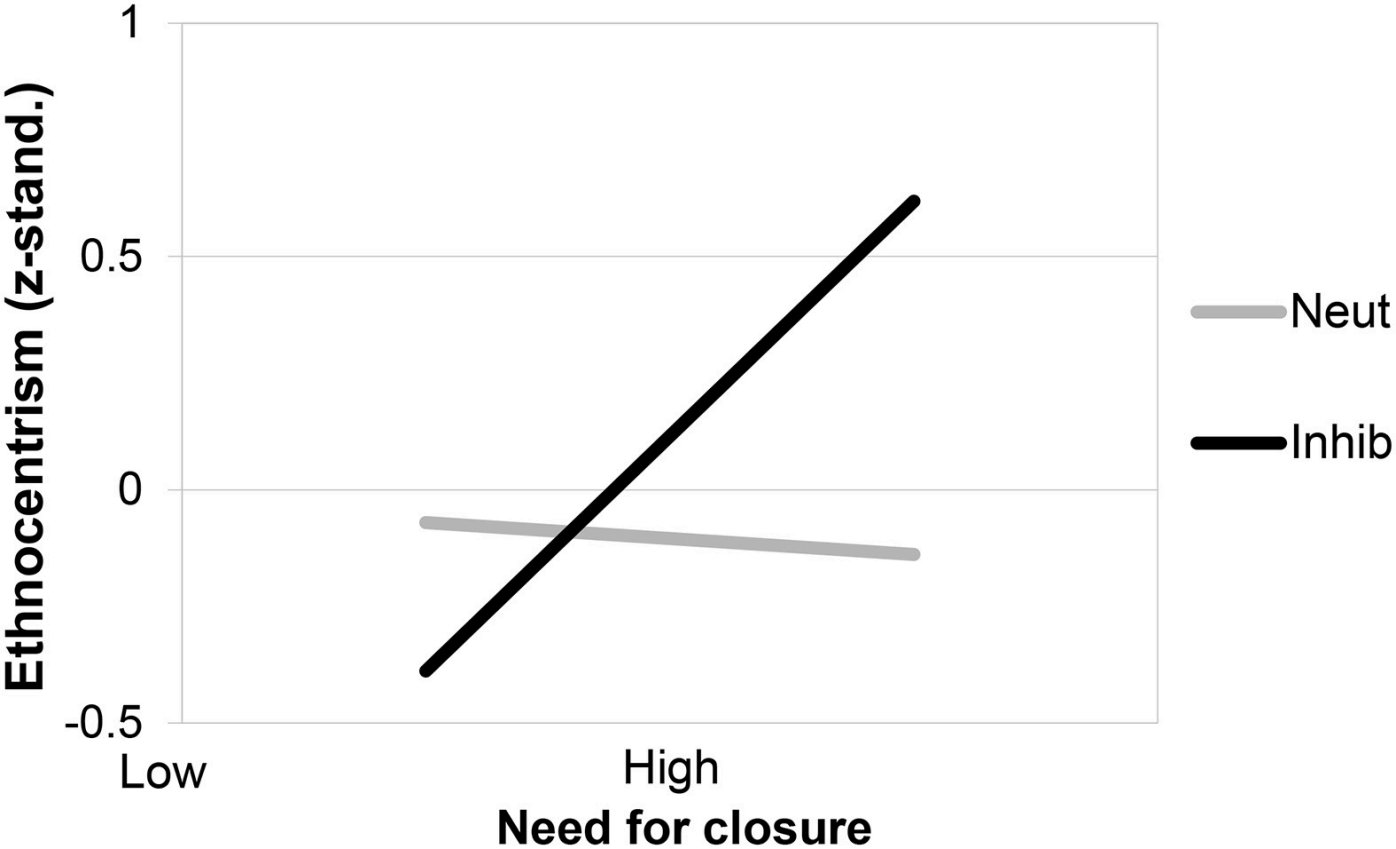
**Ethnocentrism as a function of need for closure (NFC) and Inhibition (Inhib)**. Neut, neutral control topic.

To examine whether high NFC persons respond with increased ethnocentrism to the inhibition recall-task, we used moderated regression analyses^18^. Whereas, there were no significant main effects (*p*s > 0.14), the interaction between the manipulation (coded as inhibition = 1, control = 0) and NFC was significant, *b* = 0.49, *SE* = 0.21, β = 0.27, *t*_(69)_ = 2.38, *p* < 0.02, Δ*R*^2^ = 0.07 (see Figure 6). As predicted, high NFC individuals (*SD* = 1) showed significantly increased ethnocentrism following MS, *b* = 0.59, *SE* = 0.24, β = 0.39, *t*_(69)_ = 2.41, *p* < 0.05, whereas this was not true for low NFC persons (*SD* = −1), *b* = −0.24, *SE* = 0.25, β = −0.16, *t*_(69)_ = −0.98, *p* = 0.33. Thus, our hypotheses were corroborated: inhibition caused ethnocentrism in high NFC persons, whereas low NFC persons were not affected by inhibition in this way.

#### BIS affect (manipulation check)

As expected, BIS affect was significantly higher in the inhibition condition (*M* = 3.63, *SD* = 1.24) than in the control condition (*M* = 1.32, *SD* = 0.47), *t*_(35.40)_ = 9.65, *p* < 0.001, η^2^ = 0.63^19^.

#### PANAS

Mood was also affected by inhibition. Negative affect was significantly increased and positive affect was significantly decreased by inhibition, *t*s > 2.91, *p*s < 0.01. Therefore, we reanalyzed the inhibition effect on BIS affect including the PANAS scales as covariates. Inhibition still increased BIS affect notwithstanding the PANAS, *F*_(1, 65)_ = 49.64, *p* < 0.001, η^2^ = 0.43, although BIS affect was highly correlated with negative mood, *r* = 0.79, *p* < 0.001. There was no correlation between BIS affect and positive mood, *r* = −0.16, *p* = 20.

Likewise, the interactive effect of inhibition and NFC on ethnocentrism was virtually unaltered after controlling for the PANAS subscales, *b* = 0.44, *SE* = 0.22, *t*_(63)_ = 1.95, *p* = 0.051. Persons with high NFC still showed increased ethnocentrism after inhibition, *b* = 0.67, *SE* = 0.31, *t*_(63)_ = 2.14, *p* < 0.05, contrary to low NFC persons, *b* = −0.07, *SE* = 0.29, *t*_(63)_ = −0.26, *p* = 0.80. The PANAS scales did not affect ethnocentrism (*p*s > 0.21).

#### Exploratory analyses: BIS affect × NFC on ethnocentrism

**Figure 7:**
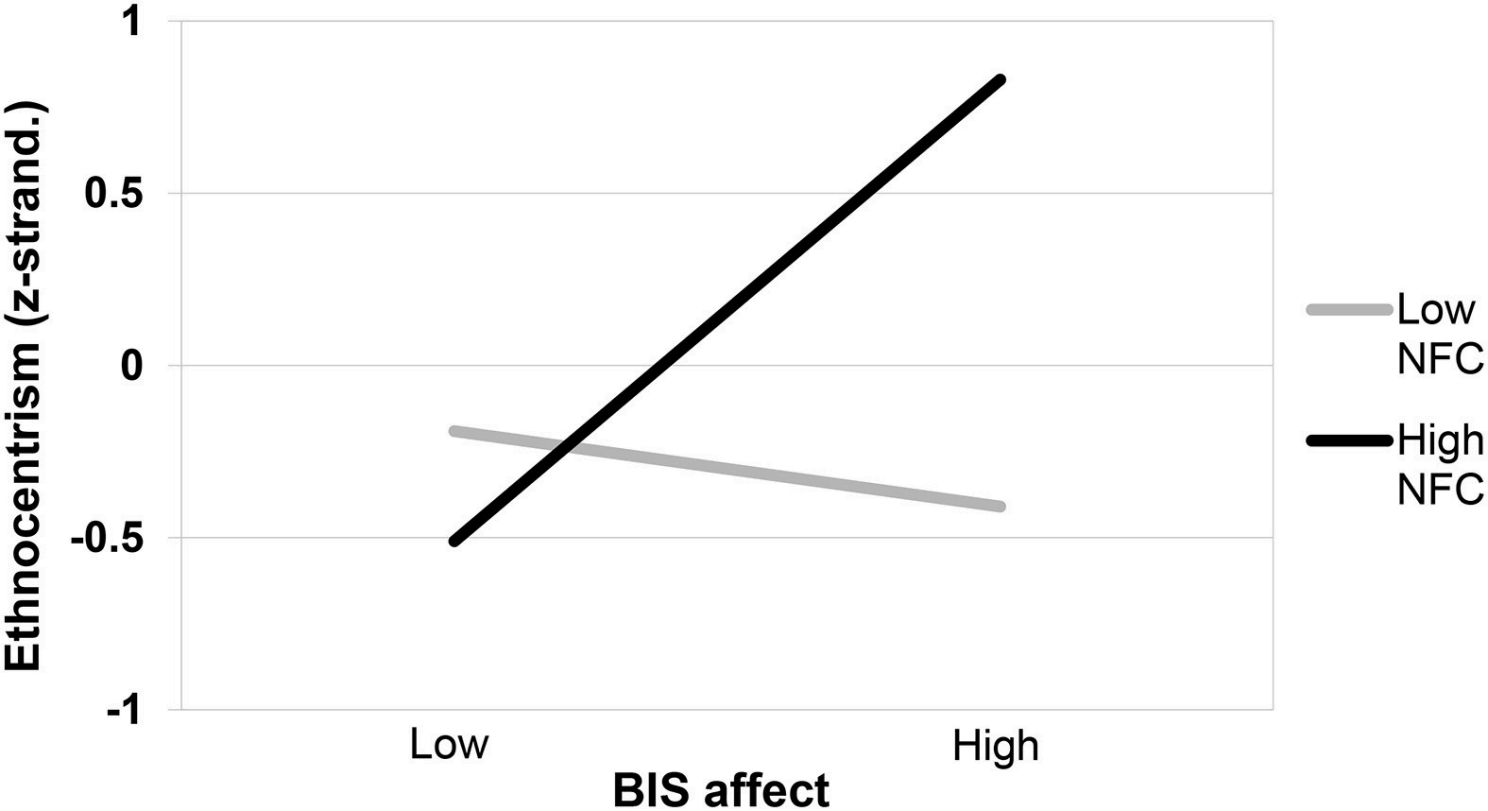
**Ethnocentrism as a function of need for closure (NFC) and BIS affect**. BIS, behavioral inhibition system.

For the sake of convergent validity, we explored whether BIS affect elicited ethnocentrism for high NFC persons similar to the inhibition manipulation. We also controlled for the PANAS scales to rule out the possibility that the effect of BIS affect could be explained by mood more generally. There was a marginally significant main effect of BIS affect, *b* = 0.17, *SE* = 0.10, β = 0.32, *t*_(65)_ = 1.69, *p* < 0.10, all other *p*s > 0.22. Moreover, a significant interaction between BIS affect and NFC occurred, *b* = 0.24, *SE* = 0.09, β = 0.39, *t*_(65)_ = 2.73, *p* < 0.01, Δ*R*^2^ = 0.09 (see Figure 7). As predicted, high NFC individuals showed significantly increased ethnocentrism following MS, *b* = 0.36, *SE* = 0.12, β = 0.66, *t*_(65)_ = 3.04, *p* < 0.01, whereas this was not true for low NFC persons, *b* = −40.05, *SE* = 0.13, β = −0.11, *t*_(65)_ = −0.43, *p* = 0.67. Again, the PANAS scales did not affect ethnocentrism (*p*s > 0.21). Thus, BIS affect evoked ethnocentrism for high NFC persons in the same way as inhibition, and this effect was independent of changes in mood.

### Discussion

These results constitute an important conceptual replication of the relationship between BIS activity and ethnocentric thinking. Manipulating inhibition, the proposed mediator of MS effects on ethnocentrism, provided further support for the proposition that inhibition plays a causal role in generating ethnocentrism following MS. Persons with high NFC responded with increased ethnocentrism to not only induced inhibition but also experiences of BIS-related affect. These results provide important multimethod evidence for the notion that not only perceptual and neural (Studies 1–2) but also cognitive-affective indicators of BIS-mediated inhibition can provoke ethnocentric thinking, and these effects are particularly strong for persons with high NFC.

## General discussion

In this paper, we have presented evidence from three studies that shed light on several previously uninvestigated questions that have recently gathered much attention in the existential threat literature (Tritt et al., 2012; Jonas et al., 2014). We found that (a) MS causes BIS activity, as indexed by right-hemispheric asymmetric activation in the frontal cortex (measured by the LBT in Study 1 and EEG in Study 2), (b) BIS activity mediates MS effects on cultural closed-mindedness (operationalized as reluctance to explore foreign cultures in Study 1 and ethnocentrism in Studies 2 and 3), and (c) these effects are particularly strong for persons inclined to experience discomfort when faced with unfamiliar and potentially threatening stimuli (measured by NFC in all studies). In addition, Study 3 conceptually replicated the first two studies by showing that directly manipulating inhibition promotes ethnocentric thinking for high NFC persons in a manner similar to right-frontal asymmetry. To our knowledge, these results are the first to demonstrate that MS-based aversion to cultural otherness is driven by a very basic, neurally-instantiated inhibitory process.

### Theoretical implications

The findings have important implications for understanding the neural and motivational underpinnings of ethnocentric attitudes in response to existential threat. They provide the first direct support for the hypothesis that inhibitory processes, as indicated by relative right-hemispheric activity, underlie MS effects on ethnocentric thinking, which is a key aspect of worldview defense. This mediational finding is consistent with the process model of threat and defense, which states that defensive responses to threat are driven by passive avoidance motivation, for which the BIS is primarily responsible (Jonas et al., 2014). This is also in line with the assumption of TMT that ethnocentric responses to MS constitute “avoidant defenses,” which are aimed at avoiding experiencing existential anxiety (Greenberg et al., 2003, p. 519). Moreover, our results are exactly in line with Greenberg et al.'s (2003) speculation that these defenses might be mediated by “a relative increase in right-hemisphere frontal lobe activity” (Greenberg et al., 2003, p. 519). On a general level, our findings may advance and refine TMT research in that they show that MS can not only promote various BIS-related behaviors (Landau and Greenberg, 2006; Routledge et al., 2010; Huang and Wyer, 2015), but also that cultural closed-mindedness can be explained through very basic inhibitory processes.

To be clear, we are not suggesting that all manifestations of worldview defense in response to MS are driven by inhibitory processes based on the current observations. Although, this would follow from the general process model (Jonas et al., 2014), our results can only speak to cultural closed-mindedness. Thus, it largely remains an empirical question to what extent inhibition processes drive other forms of worldview defense given that inhibition has yet to be thoroughly tested as a mediator in this regard. Future research should sample the conceptual space of worldview defense more broadly to examine the generality of the process we present On a related note, it also remains to be seen whether approach-related cognition can alleviate the passive avoidance observed here and prevent negative effects of MS on desirable outcomes, like creativity (Sligte et al., 2013). The findings may also advance research on threat and defense in that they suggest that various responses to threat that are not obviously or intuitively related to a lack of approach motivation may be attributed to the approach-inhibitory activity of the BIS.

These advances also have important implications for inspiring research on the defensive function of BIS anxiety, which originates from ethological research on animals' responses to threat (Gray and McNaughton, 2000). It has been argued that “there are long-standing concerns that rodent models of psychological processes are too simple to apply in humans (Matthews, 2008): For example, there is no evidence that rodents experience anxiety of an abstract type related to existential issues, whereas historical and literary accounts abundantly point to the existence of such angst in humans. Concerns of this type have raised a need for studies of human defense that can test the validity of the defensive explanation for anxiety in humans” (Perkins and Corr, 2014, pp. 42–43). Our findings show that rodent models of BIS anxiety can indeed be applied to defensive behaviors in the wake of threat in humans and point toward the possibility of a more consilient science of motivation.

On the whole, our findings provide valuable insight into contextual, interindividual, motivational, and neural determinants of cultural closed-mindedness. To our knowledge, this research is the first to establish an empirical link between very basic, neurally instantiated inhibitory processes and rather complex, higher-order manifestations of intergroup negativity. By investigating the neural and motivational underpinnings of ethnocentric attitudes in the wake of existential threat, this research might contribute to a fuller understanding of cultural worldview defensive phenomena, among the most investigated outcomes in social psychology over the last 25 years (Burke et al., 2010).

## Limitations and future directions

We used an indirect measure of BIS activity in Studies 1 and 2. Following theoretical and empirical research relating reduced left-frontal asymmetry (or increased right-frontal asymmetry) to BIS-mediated inhibition of approach motivation, we used perceptual and neural indicators of frontal asymmetry to gauge BIS activity (e.g., Davidson et al., 2000; Coan and Allen, 2004; Corr, 2004; Harmon-Jones et al., 2009; Nash et al., 2010). To provide more direct evidence for the role of inhibition in the emergence of ethnocentric thinking, we directly manipulated inhibition in Study 3, finding increased ethnocentrism for high NFC persons following experiences of inhibition and BIS-related affect. In combination, these studies yield support for BIS-driven ethnocentrism in the wake of existential threat. Further, we have other lines of converging evidence for the notion that perceptual and neural indicators of right-frontal asymmetry reflect BIS activity (Agroskin and Jonas, 2015; Agroskin et al., 2015). For instance, we have found in four EEG and LBT studies that directly boosting/blocking BIS anxiety leads to increased/reduced right-frontal asymmetry following MS (and also without MS). Mirroring increased BIS affect following induced inhibition (Study 3), we have also found amplified BIS affect following MS. In summary, accumulating, multi-method evidence supports the notion that threat-induced anxious inhibition (BIS activity) is a cause of ethnocentric thinking.

Future research might consider an intriguing existential interpretation of the avoidance—approach motivational systems. According to TMT, avoidance—approach conflicts may reflect the duality between the core motives for self-protective defense (avoidance motivation) and self-expansive growth (approach motivation; Greenberg et al., 1995a; Pyszczynski et al., 2000, 2012). From this perspective, closed-minded persons engage in novelty-avoidant defense of the “familiar” in response to mortality awareness, whereas open-minded individuals approach the “unfamiliar,” expanding the self by gaining new experiences. Future research may investigate to what extent these motivational tendencies mediate MS effects on various behavioral manifestations of the defense and growth motives, such as support of extreme military action against cultural outgroups (defense motive), and commitment to important life goals that convey a sense of existential meaning (growth motive; Greenberg et al., 1995a; Pyszczynski et al., 2012; see Vail et al., 2012 for a review of growth-oriented MS effects).

Another avenue for future research might be to investigate further neural underpinnings of ethnocentric attitudes following MS. Along with the right dorsolateral prefrontal cortex, the BIS may be subserved by the anterior cingulate cortex (ACC; McNaughton and Corr, 2004). In addition to generating neural signals of error-related distress (ERN) and anxiety (see Shackman et al., 2011 for a review), the ACC contributes to risk aversion in decision making (Brown and Braver, 2007). This suggests that the ACC may also support risk-avoidant behaviors after MS, such as the ethnocentric avoidance of cultural outgroups. Future research might shed some more light on previously unexamined neural mediators of terror management mechanisms.

## Conclusion

Becker (1997) saw man as a “terrified, death-avoiding animal” (p. 99) that, by seeking to avoid death, kills off “so large a spectrum of his action-world that he is actually isolating and diminishing himself and becomes as though dead” (Becker, 1997, p. 181). Psychiatrist Joseph Rheingold arrived at the same conclusion: “The common denominator of all negative ways of dealing with anxiety is a shrinking of the area of awareness and of activity. (…) We are afraid to die, and therefore we are afraid to live, (…) we avoid non-being by avoiding being. The avoidance of anxiety then means a kind of death in life” (Rheingold, 1967, pp. 204–205). These insightful analyses shed light on a fascinating irony inherent in human striving for self-preservation, or in terms of terror management, a life free from consciously experienced death anxiety (Greenberg et al., 2003). In trying to avoid death, people actually avoid life, given that exploring (cultural) novelty contributes to various indicators of well-being, including subjective vitality and satisfaction with life (Kashdan et al., 2004). Our findings represent the first neural evidence, to our knowledge, that this existential ironical twist is driven by an ancient motivational system, oriented toward passively avoiding punishment from the environment. By symbolically avoiding the punishment of death, people in reality obtain the punishment of an unlived life.

## Author contributions

DA and EJ conceptualized the project and collected the data. JK advised the EEG data collection and analysis and assisted manuscript preparation. DA analyzed the data and wrote the manuscript. MP assisted with data analysis and manuscript preparation.

## Funding

The research reported in this article has been financed by the Austrian Science Fund (FWF-P27457). The first author of this article was also financially supported by the Doctoral College “Imaging the Mind” of the Austrian Science Fund (FWF-W1233).

### Conflict of interest statement

The authors declare that the research was conducted in the absence of any commercial or financial relationships that could be construed as a potential conflict of interest.

## Footnotes

^1^Corr et al. (2013a) propose a distinction between a system for *passive* avoidance, generated by the BIS (e.g., inhibition of approach motivational behavior toward unfamiliar and potentially threatening people or places), and a system for *active* avoidance, produced by the fight-flight-freeze system (FFFS; e.g., running away from a predator). Contrary to the BIS that responds to more distant, abstract, and ambiguous threats, the FFFS responds to more proximate, concrete, and unequivocal threats, such as immediate attackers. The present line of research pertains to existential threats, which are more distant, abstract, and ambiguous (e.g., the time and way of one's death is usually unknown; see also Huang and Wyer, 2015). Therefore, we focus on BIS-mediated *passive* avoidance motivational responses to threat in this paper.

^2^In some studies discussed in this paper, NFC was measured with the personal need for structure (PNS) scale (Neuberg and Newsom, 1993), which has been used as an alternative operationalization of NFC in prior TMT research (e.g., Dechesne et al., 2000).

^3^Regarding sample size considerations, MS has an expected effect size of MS at *d* = 0.75 on ethnocentrism and related outcomes (Burke et al., 2010). Thus, power for a main effect is at 80% with a sample of *n* = 60. Given that a personality moderator should add prediction to a model, power was expected to be well over 80% for the current studies. For example in the current research domain, Vess et al. (2009) observed NFC × MS interaction effects on meaning in life in three studies (1a–1c) with an average *d* = 0.90.

^4^In contrast to Vess et al. (2009) who used Green and Campbell's (2000) exploration scale, we developed new items for cultural novelty-avoidance because their exploration scale largely lacks explicit reference to culturally foreign stimuli.

^5^Due to the intensive nature of EEG data collection and analysis, collection was stopped after reaching an *n* of 15 per cell plus 10% for anticipated data loss.

^6^Age was unequally distributed across experimental conditions. Participants were significantly younger in the MS condition (*M* = 20.4, *SD* = 1.93) than in the control condition (*M* = 23.7, *SD* = 2.91), *t*_(31)_ = 3.78, *p* < 0.001. We included age as a covariate in all analyses reported below. Running the analysis without this covariate did not impact the status of the significance tests or the substance of the conclusions.

^7^This study was part of a larger project that included other experimental conditions for exploratory reasons. Specifically, conditions not reported here were presented with other poems about existential topics that did not refer to the subject of death and that were not designed as neutral control conditions either. Given that this study was primarily designed as a conceptual replication of Study 1, we present only the analyses that concern the MS and neutral control conditions.

^8^In addition to the 33 participants reported above, nine more persons participated in the EEG study but failed to complete the personality questionnaires. They were dropped from all analyses.

^9^Two items were dropped prior to analyses because of very low corrected item-total correlations (*r*_*it*_s < 0.20).

^10^The press reports described crimes committed in Austria (or by Austrians). Then, participants' intentions to punish the perpetrators were measured. Given that 66.7% of our participants were Germans, we could not analyze their punishment intentions in response to Austrian crimes as indicators of their ethnocentric, worldview-defensive attitudes (cf. Rosenblatt et al., 1989). We also included four items measuring worldview-defensive prejudice toward immigrants (Agroskin and Jonas, 2010) right after the focal ethnocentrism measure (see below), which were not analyzed for the same reason (i.e., most of our participants were immigrants themselves).

^11^Two items were dropped prior to analyses because of very low corrected item-total correlations (*r*_*it*_s < 0.20).

^12^Two participants mentioned death-related thoughts and ethnocentric attitudes, though. Dropping these two participants did not substantially change the results reported below, which is why we kept them in the final sample.

^13^Four participants had missing data in either the proximal or the distal EEG measurement. All reported analyses have been run with the whole sample using Full-Information-Maximum Likelihood (FIML) parameters in Mplus 7 (Muthén and Muthén, 2012). However, excluding the participants with missing data using listwise deletion did not change the results substantially (see also footnote ^15^).

^14^All critical effects stayed significant using non-bootstrapped coefficients, including the conditional indirect effect for high NFC, *b* = 0.69, *SE* = 0.33, *t* = 2.10, *p* < 0.05. However, although the point estimate for the indirect effect was even somewhat increased, the bootstrap estimates became less robust, *b* = 0.69, 90% CI [0.05, 1.54], 95% CI [−0.11, 1.73]. This may be due to reduced degrees of freedom. Note, that the same pattern occurred when computing the model without the PANAS using listwise deletion instead of FIML, which likewise reduced degrees of freedom, *b* = 0.62, *SE* = 0.31, *t* = 2.04, *p* < 0.05 (non-bootstrap test), *b* = 0.62, 90% CI [0.04, 1.23], 95% CI [−0.11, 1.34] (bootstrap test; see also footnote 16). The results were virtually unchanged after controlling for the PANAS scales.

^15^With respect to the stopping rule for data collection in this study, we aimed at recruiting the same amount of participants as in Study 1, distributing questionnaires to all students who attended a lecture that is usually attended by about 80 people.

^16^After the ethnocentrism items, we included four items measuring prejudice toward immigrants (Agroskin and Jonas, 2010). These items were included as another measure of an ethnocentric, worldview-defensive attitude but were not analyzed because 75% of participants of this study were technically immigrants themselves (i.e., Germans studying in Austria).

^17^The negative mood scale included only eight instead of ten items because the items *anxious* and *nervous* were included in the BIS affect measure.

^18^Three participants failed to complete the ethnocentrism measure.

^19^Seven participants failed to complete the BIS affect and PANAS measures. We also used the adjusted degrees of freedom because variances were not equal as indicated by a significant Levene's test (*p* < 0.001).
